# Whole-genome sequencing characterization of silver-resistant bacteria from the outfall of wastewater treatment plants and effluent-receiving rivers

**DOI:** 10.1128/aem.00022-25

**Published:** 2025-08-05

**Authors:** Yubing Xia, Haichen Wang, Jun Li, Haolan Wang, Yuyao Wang, Yongmei Hu, Fengjun Xia, Mingxiang Zou

**Affiliations:** 1 National Clinical Research Center for Geriatric Disorders, Xiangya Hospital, Central South University 12570, Changsha, Hunan, People’s Republic of China; 2 Department of Clinical Laboratory, Xiangya Hospital, Central South University 616579, Changsha, Hunan, People’s Republic of China; Colorado School of Mines, Golden, Colorado, USA

**Keywords:** silver resistance, whole-genome sequencing, *sil *operon, wastewater

## Abstract

The excessive use of silver compounds has led to the environmental dissemination of silver resistance genes. However, little is known about the epidemiology of silver-resistant bacteria in the environment. Wastewater treatment plants (WWTPs) link the clinical settings with the natural environment and serve as a major pathway for silver entering the natural environment. However, their role in the dissemination of silver resistance genes remains unclear. This study investigated the characteristics of silver-resistant bacteria in the vicinity of four WWTPs to assess their environmental impact. Water and sediment samples were collected from the WWTP outfalls and downstream rivers. Among 22 silver-resistant strains obtained through plate screening, the majority were *Klebsiella* spp., followed by *Escherichia* spp. and *Kluyvera* spp. Notably, ST23 and ST2464 were the predominant sequence types (multilocus sequence typing) identified among the *Klebsiella pneumoniae* isolates. Antimicrobial susceptibility testing and whole-genome sequencing were performed to identify environmental heavy metal and antibiotic-resistant genes. Whole-genome sequencing revealed the presence of the *sil* and *pco* operons, which together formed the copper homeostasis and silver resistance island. The silver resistance gene sequences varied. Various heavy metal resistance genes, including *mer* and *ars*, were detected in the strains, as were a diverse array of plasmid types, including IncFIB(K) and repB(R1701), and *fosA* and *β*-lactamase encoding genes. Taken together, the findings underscore the coexistence of silver resistance genes with multiple heavy metal resistance genes in wastewater bacteria, highlighting the environmental implications of silver usage. Efforts should be directed toward restricting silver usage, improving WWTP purification methods to safeguard human and environmental health.

**IMPORTANCE:**

The misuse of silver compounds has led to an increasing presence of silver-resistant microorganisms in the environment, which cannot be completely eliminated in wastewater treatment plants, allowing them to enter the environment and pose risks to environmental safety and human health. However, research on the epidemiology of silver-resistant bacteria in wastewater and their whole-genome sequencing remains limited. Our findings explain that silver-resistant bacteria from the environment often possess resistance to other heavy metals, share genetic similarities, and possess the potential for widespread transmission. Furthermore, these bacteria may enter clinical settings through environmental pathways, posing a risk to human health.

## INTRODUCTION

In recent years, the misuse and overuse of antibiotics have led to the rise of antibiotic-resistant bacteria, prompting researchers to explore non-antibiotic compounds for combating bacterial infections (1, 2). Among these promising non-antibiotic compounds, silver has attracted considerable attention due to its potent and broad-spectrum antimicrobial activity. This metal element can be utilized for the control of bacterial infections in various forms, including silver ions and silver nanoparticles (3). The antimicrobial effects of silver ions involve disruption of bacterial membranes and inhibition of bacterial enzyme activities. Silver nanoparticles, with their large surface area and unique physicochemical properties, can interact more effectively with bacteria (4). Consequently, these forms of silver are being increasingly used in clinical settings. Unfortunately, research data have demonstrated that the growing utilization of silver materials has led to the gradual emergence of silver resistance, raising significant concerns. This resistance is not confined to specific bacteria, but rather involves diverse bacterial species, garnering widespread attention from both scientific and medical communities (5). Clinically, there have been numerous instances of silver-resistant bacteria isolated from wounds. Bacterial pathogens harboring the *sil* genes have been identified (6, 7). These findings suggest a diminishing effectiveness of silver as an antimicrobial agent, with the distribution of the *sil* genes becoming increasingly widespread.

Silver resistance in bacteria primarily involves the combination of efflux pumps and silver-binding proteins to restrict silver from entering the cytoplasm (8). The *sil* operon, found in the earliest identified silver-resistant plasmid of *Salmonella enterica serovar Typhimurium* (pMG101), plays an important role in exogenous silver resistance (9). The *sil* operon contains genes encoding efflux pumps (*silCBA* and *silP*), a silver-binding protein gene (*silE*), and regulatory genes (*silS* and *silR*). The *sil* operon and *pco* operon are often located on plasmids and compose the copper homeostasis and silver resistance island (CHASRI) (10). Endogenous silver resistance has also been reported to be due to the *cus* operon. The *cus* operon also includes genes for a sensor kinase (CusS), a periplasmic efflux transporter (CusBCA), and a periplasmic Ag^+^-binding protein (CusF) (8). The two systems are thought to function primarily via silver efflux. The *copA* and *copB* genes encoding copper pump ATPases also contribute to silver resistance (11).

Wastewater treatment plants (WWTPs) are intermediaries between human society and the natural environment. Several studies have documented the presence of antibiotic resistance genes in bacteria from urban wastewater, revealing that the heavy metal pressure selective environment in WWTPs acts as a reservoir for multi-resistant bacteria (12, 13). Additionally, increased concentrations of silver nanoparticles in wastewater influence the levels of antibiotic resistance genes within wastewater systems, selectively enhancing the presence of resistance genes in prokaryotic communities (14). The reuse of treated wastewater may pose both environmental and health risks to humans. Ongoing research is crucial in developing more effective strategies to mitigate the spread of heavy metal tolerance and antibiotic resistance genes and to reduce environmental contamination resulting from wastewater discharges.

Previous studies have mainly focused on the abundance of silver-resistant bacteria isolated from clinical settings and the presence of *sil* genes in hospital and urban wastewater. However, there is a lack of research on the epidemiological characteristics of silver-resistant bacteria in wastewater and whole-genome sequencing (WGS) information. We previously revealed that Ag^+^ and AgNPs can change the microbial composition and functional pathways (15). Further investigation is needed into the microbial characteristics affected by heavy metals in wastewater environments.

In this study, we collected water and sediment samples from WWTP outfalls and downstream rivers. Twenty-two strains of silver-resistant bacteria were isolated from these samples. Their epidemiological characteristics were determined to identify the genes carried by these bacteria and their potential transmission processes, as well as to predict the control and usage of antimicrobial drugs. The findings provide important evidence and theoretical support for controlling wastewater discharge and reducing environmental pollution.

## MATERIALS AND METHODS

### Sample collection

**Fig 1:**
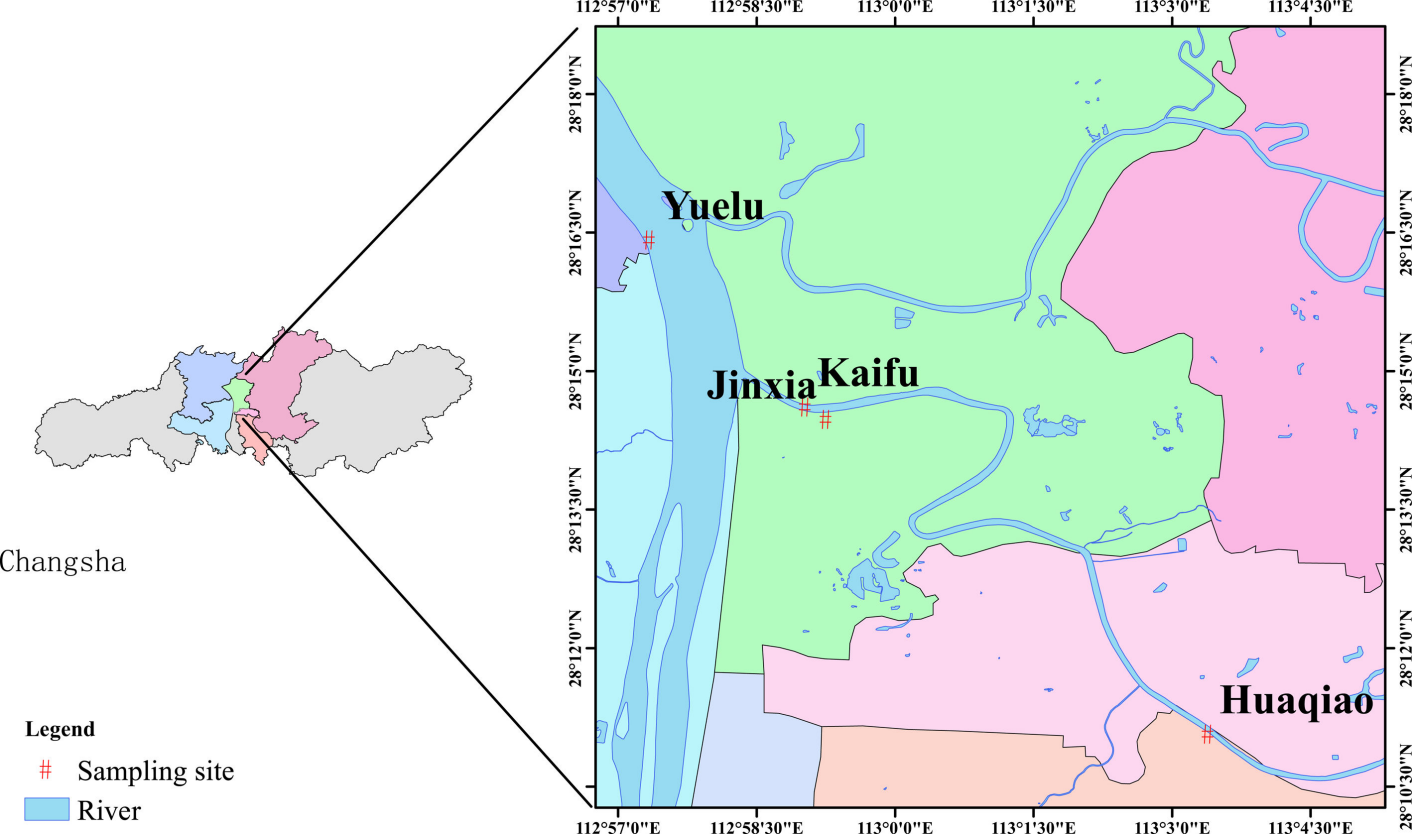
Map depicting the sampling sites.

Samples, including WWTP effluents, water from effluent-receiving rivers, and activated sludge, were collected from outfalls of four WWTPs and their downstream rivers. The Kaifu, Jinxia, Yuelu, and Huaqiao WWTPs, located in Changsha, Hunan Province, China (Fig. 1), have a wastewater treatment capacity of 300,000, 300,000, 180,000, and 160,000 m^3^/day, respectively. They handle domestic sewage from different areas of Changsha. Samples were collected from August 2022 to July 2023, with a total of 21 collections. Among them, the wastewater from the Jinxia, Kaifu, and Huaqiao WWTP discharges into the Liuyang River, whereas the YueLu WWTP’s effluent flows into the Xiangjiang River. Downstream sampling points were positioned approximately 50 m from the discharge outlets. At each sampling site, three 500 mL water samples were taken consecutively as a representative grab sample set for further analysis. Sludge samples were collected in 50 mL centrifuge tubes (three samples per site). The samples were mixed evenly after being collected. The wastewater and solid samples were collected using sterile amber-colored containers and transported on ice to the laboratory for analysis within 2 hours.

### Screening for silver-resistant bacteria

Water samples were filtered through gauze and centrifuged at 3,000 × *g* for 10 minutes at 4°C. The supernatants were removed, and each pellet was thoroughly resuspended in PBS buffer and allowed to settle naturally. Sludge samples were added to PBS and mixed completely. The potential silver-resistant strains were isolated by plating solutions on Mueller-Hinton (MH) agar supplemented with 128 µg/mL silver nitrate. Taxonomic identification of isolates was performed using the Microflex matrix-assisted laser desorption/ionization time-of-flight mass spectrometry system (Bruker Daltonik, Bremen, Germany). All strains were stored at −80°C for further analysis.

### Testing of antimicrobial susceptibility to silver nitrate and antibiotics

According to Clinical and Laboratory Standards Institute (CLSI) guidelines, the broth microdilution method was adopted to determine the minimum inhibitory concentration (MIC) to silver nitrate of all strains with Mueller-Hinton broth (MHB). Bacteria with MIC ≥ 512 µg/mL were considered resistant to silver ions (Ag^+^) (16).

The broth microdilution method was used to determine antimicrobial susceptibility against tigecycline (TGC), meropenem (MEM), colistin (COL), amikacin (AK), cefepime (FEP), ciprofloxacin (CIP), ceftazidime (CAZ), piperacillin/tazobactam (TZP), cefoxitin (FOX), imipenem (IPM), ceftriaxone (CRO), levofloxacin (LEV), trimethoprim/sulfamethoxazole (SXT), gentamicin (CN), and aztreonam (ATM). Tigecycline results were interpreted by the European Committee on Antimicrobial Susceptibility Testing breakpoints. All other results were interpreted by CLSI breakpoints. *Escherichia coli* ATCC25922 and *Pseudomonas aeruginosa* ATCC27853 were used as quality controls.

### WGS and bioinformatic analysis

Genomic DNA was extracted using the Ezup Column Bacteria Genomic DNA Purification Kit (Sangon Biotech, Shanghai, China) according to the manufacturer’s recommendation. Fragmented DNAs were end-repaired, A-tailed, adapter-ligated, and amplified using the Next Ultra DNA Library Prep Kit for Illumina (New England Biolabs, Ipswich, MA, USA). A 150 bp paired-end sequencing run was performed on the HiSeq platform (Illumina, San Diego, CA, USA) previously. After trimming the raw reads, the sequences were assembled *de novo* using SPAdes (version 3.1.1) (17). Draft genomes were annotated using Prokka (version 1.10) and RAST annotation software (18, 19). Species confirmation of isolates was identified using Genome Taxonomy Database-Toolkit (GTDB-TK) software (20). Gene contamination was detected using CheckM software (21). Heavy metal resistance genes were identified using BacMet (Antibacterial Biocide and Metal Resistance Genes Database) (22). The whole-genome sequencing failed to annotate the *sil* and *pco* genes in a particular strain, which were supplemented using PCR. The *sil* operon, including *silRS*, *silE*, *silCBA*, *silP*, and *silF*, and the *pco* operon, including *pcoA*, *pcoB*, *pcoC*, *pcoD*, *pcoE*, *pcoR*, and *pcoS*, were analyzed. The primers used are listed in Table S1. PCR products were electrophoresed using 1.2% agarose gel electrophoresis and visualized using ultraviolet light under a transilluminator. Antibiotic resistance genes and types of plasmids were identified by ResFinder and PlasmidFinder, respectively, from the Centre for Genomic Epidemiology (23, 24). *In silico* analyses of the sequence type (ST) of the strains were performed via multilocus sequence typing using MLST 2.0 (25). To assess the homology among strains, the core-genome tree was constructed using Roary (26).

## RESULTS

### Isolation of multiple silver-resistant bacteria from the environments of the WWTPs

A total of 22 strains of silver-resistant bacteria were isolated from the environmental samples collected from August 2022 to July 2023. The strains belonged to *Klebsiella pneumoniae* (*n* = 13), *Klebsiella variicola* (*n* = 1), *E. coli* (*n* = 3), *Kluyvera ascorbata* (*n* = 2), *Enterobacter cloacae* (*n* = 1), *Enterobacter asburiae* (*n* = 1), and *Citrobacter freundii* (*n* = 1) genera (Table S2). Among the 22 isolates, 18 originated from water samples and 4 strains from sediment samples.

### Antibiotic susceptibility profiles of silver-resistant strains

Based on cut-off MIC values >512 µg/mL for silver resistance, all 22 recovered isolates exceeded this threshold. Among the tested strains, the highest resistance rate was detected for trimethoprim/sulfamethoxazole (22.7%; 5/22), followed by ciprofloxacin (9.1%; 2/22) (Table 1). Only one strain (620; *Kluyvera ascorbata*) was resistant to colistin. However, resistance to the other antibiotics was not phenotypically evident in any of the tested strains (Table S3).

**TABLE 1:** MIC distribution of isolates (*n* = 22) from WWTPs^*a*^

Antimicrobial agent	Breakpoint (μg/mL)	Isolates (%)														Resistant (%)
		0.125	0.25	0.5	1	2	4	8	16	32	64	128	256	512	>512	
Silver nitrate	>512	–	–	–	–	–	–	–	–	–	–	–	–	–	100	100
TGC	≥8	–	13.6	59.1	27.3	–	–	–	–	–	–	–	–	–	–	0
COL	≥4	–	4.5	50.0	41.0	–	–	–	4.5	–	–	–	–	–	–	4.5
CIP	≥1	86.4	4.5	–	9.1	–	–	–	–	–	–	–	–	–	–	9.1
MEM	≥4	100	–	–	–	–	–	–	–	–	–	–	–	–	–	0
FOX	≥32	–	–	–	–	15.8	52.6	10.5	21.1	–	–	–	–	–	–	0
FEP	≥16	90.9	9.1	–	–	–	–	–	–	–	–	–	–	–	–	0
IPM	≥4	40.9	45.5	9.1	–	4.5	–	–	–	–	–	–	–	–	–	0
CAZ	≥16	50.0	31.8	18.2	–	–	–	–	–	–	–	–	–	–	–	0
CRO	≥4	77.3	9.1	9.1	–	4.5	–	–	–	–	–	–	–	–	–	0
LEV	≥2	63.6	9.1	18.2	9.1	–	–	–	–	–	–	–	–	–	–	0
AK	≥64	–	–	–	–	–	77.3	22.7	–	–	–	–	–	–	–	0
SXT	≥4/76	22.7	13.6	36.4	4.5	–	22.7	–	–	–	–	–	–	–	–	22.7
TZP	≥128/4	–	–	–	31.8	36.4	31.8	–	–	–	–	–	–	–	–	0
CN	≥16	–	–	4.5	90.1	–	4.5	–	–	–	–	–	–	–	–	0
ATM	≥16	100.0	–	–	–	–	–	–	–	–	–	–	–	–	–	0

^*a*^ Vertical lines indicate clinical cut-off values. The counts have been adjusted to exclude bacteria that are naturally resistant to the antibiotic. The gray-shaded areas indicate values that exceed the detectable dilution range. MIC values greater than the highest concentration in the range are presented as one dilution step above the range. – indicates that no isolates were observed to have this concentration as their MIC value.

### Analysis of resistance genes

**Fig 2:**
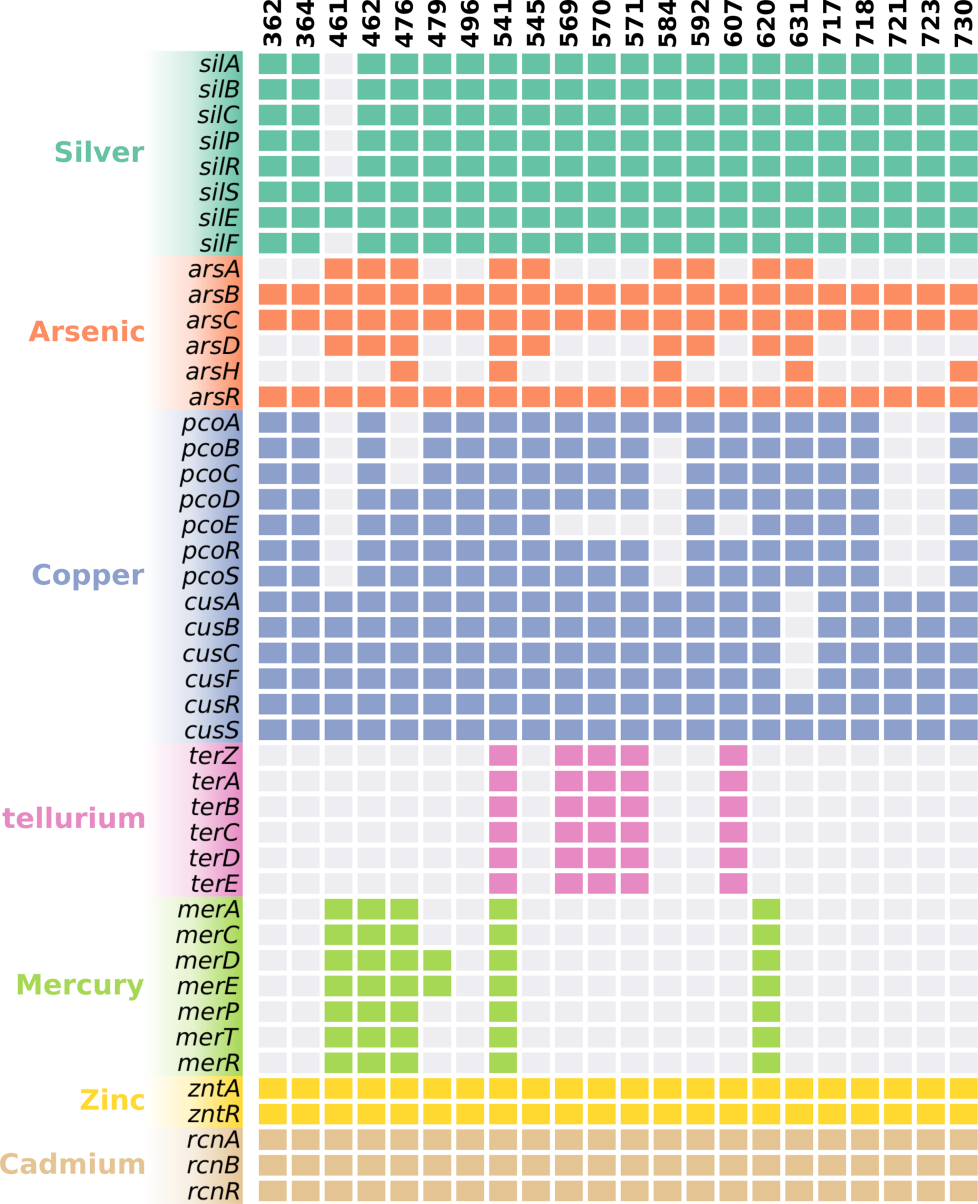
Heavy metal resistance genes among isolates. The colored squares mean that gene is present.

WGS analysis of the 22 strains was performed to reveal their genetic backgrounds. The analysis revealed that all the environmental silver-resistant isolates, except strain 461, carried the entire *sil* operon with *silS*, *silR*, *silC*, *silA*, *silB*, *silE*, *silF*, and *silP* (Fig. 2). Furthermore, 19 strains with the *sil* operon carried the *pco* operon, both of which together formed the copper homeostasis and silver resistance island. In strain 461, only *silE* and *silS* were detected using WGS. However, PCR detected the entire *sil* and *pco* operons in strain 461 (Fig. S1). WGS results showed that in strain 584, only *pcoA* was detected, while strain 476 lacked *pcoA*, *pcoB*, and *pcoC*, and four strains (569, 570, 571, and 607) lacked *pcoE*. These results were also identified with PCR analysis. The sequences of *silS*, *silR*, and *silE* were compared with the corresponding region of pMG101 (GenBank no. AF067954; Table 2). *silR* showed sequence variations between 8.30% and 1.31% at the nucleotide level. Compared to the *silR* gene of pMG101, *silR* of strains 584 and 730 featured a deletion of 6 bp. Only strain 632 displayed near-identity (99.07%) of *silE* with the corresponding gene in pMG101. The *silE* genes in strains 584 and 730 exhibited a high degree of divergence from the *silE* gene in pMG101, with a respective similarity of 82.45% and 82.69%, while the variation in the remaining strains was 9.49%–3.24% at the nucleotide level. The *silS* gene in isolated strains varied by 5.99%–3.97%. Additionally, except for strain 631, WGS results indicated that all strains carried the *cusABFCRS* genes.

**TABLE 2:** Overall identities of *sil* genes compared with pMG101

Strain	*silE* (%)	*silS* (%)	*silR* (%)
362	91.69	94.28	93.01
364	91.69	94.35	93.01
461	91.92	94.91	–^*a*^
462	91.92	94.35	93.01
476	92.15	94.28	91.70
479	96.53	95.00	98.69
496	91.92	94.35	93.01
541	92.84	94.35	92.87
545	96.76	94.35	93.01
569	90.51	94.15	92.58
570	90.51	94.22	92.58
571	90.51	94.22	92.58
584	82.45	95.90	91.85
592	96.06	94.98	93.89
607	90.51	94.51	92.58
620	91.22	94.69	91.99
631	99.07	95.31	97.23
717	91.92	94.01	98.69
718	91.92	94.01	98.69
721	91.69	94.28	92.87
723	91.69	94.28	92.87
730	82.68	96.03	91.85

^*a*^ –, no sequence alignment data available.

WGS revealed the presence of genes encoding efflux system proteins, including *sugE*, and the presence of genes associated with stress regulation, such as *rpoS*, in all the strains. Moreover, a variety of heavy metal resistance genes were identified across multiple strains (Fig. 2). All isolates featured the presence of the *ars* and *znt* genes, which confer resistance to arsenic and zinc, respectively. Strains 541, 569, 570, 571, and 607 harbored the complete *ter* operon, associated with resistance to tellurium. Moreover, strains 461, 462, 476, 541, and 620 harbored the entire *mer* operon with *merACDEPTR*, providing resistance to mercury, whereas strain 479 contained only the *merD* and *merE* genes. The prevalent antibiotic resistance gene was *fosA* (*n* = 10). Various *β*-lactamase encoding genes were detected, including *bla*_TEM-1B_ (*n* = 2), *bla*_LEN3_ (*n* = 3), *bla*_CMY-75_ (*n* = 1), *bla*_SHV-99_ (*n* = 1), *bla*_ACT-15_ (*n* = 1), *bla*_SHV-27_ (*n* = 1), *bla*_SHV-190_ (*n* = 4), *bla*_LAP-2_ (*n* = 1), *bla*_SHV-148_ (*n* = 1), *bla*_CTX-M-9_ (*n* = 1), *bla*_OKP-B-7_ (*n* = 2), *bla*_SHV-89_ (*n* = 2), and *bla*_CTX-M-78_ (*n* = 1). The rates for *oqx*, *tet*, *sul*, *aph*, *qnr*, *aad*, *cml*, and *dfr* genes were 40.9% (*n* = 9), 31.8% (*n* = 7), 22.7% (*n* = 5), 13.6% (*n* = 3), 13.6% (*n* = 3), 9.1% (*n* = 2), 9.1% (*n* = 2), and 9.1% (*n* = 2), respectively (Table 3).

**TABLE 3:** Antibiotic resistance genes and plasmid Inc type among isolates

Isolate	Antibiotic resistance gene(s)	Plasmid Inc types
362	*aadA1*, *aadA2*, *bla*_TEM-1B_, *cmlA1*, *dfrA12*, *floR*, *sul3*, *tet(B*)	IncFIB(K), IncR, IncX1, IncY
364	*aadA1*, *aadA2*, *bla*_TEM-1B_, *cmlA1*, *dfrA12*, *floR*, *sul3*, *tet(B*)	IncFIB(K), IncR, IncX1, IncY
461	*aph(3″)-Ib*, *aph(6)-Id*, *bla*_LEN3_, *tet(A*)	IncFIA(HI1), IncFIB(K), repB(R1701)
462	*aph(3″)-Ib*, *aph(6)-Id*, *bla*_LEN3_, *tet(A*)	IncFIA(HI1), IncFIB(K), repB(R1701)
476	*bla*_CMY-75_	pKPC-CAV1321, repB(R1701)
479	*bla*_SHV-99_, *oqxA*, *oqxB*	IncFIB(K), IncR
496	*bla*_LEN3_	IncFIB(K)
541	*bla*_ACT-15_, *fosA*	IncFIB(pECLA), IncFIB(pHCM2), IncFII(pECLA)
545	*bla*_SHV-27_, *fosA6*, *oqxA*, *oqxB*	IncFIB(K)
569	*bla*_SHV-190_, *fosA6*, *oqxA*, *oqxB*	IncHI1B(pNDM-MAR), repB
570	*bla*_SHV-190_, *fosA6*, *oqxA*, *oqxB*	IncHI1B(pNDM-MAR), repB
571	*bla*_SHV-190_, *fosA6*, *oqxA*, *oqxB*	IncHI1B(pNDM-MAR), repB
584	None	None
592	*aph(3″)-Ib*, *aph(6)-Id*, *bla*_LAP-2_, *bla*_SHV-148_, *floR*, *fosA*, *OqxA*, *OqxB*, *qnrS1*, *sul2*, *tet(A*)	IncFIB(K)
607	*bla*_SHV-190_, *fosA6*, *oqxA*, *oqxB*	IncHI1B(pNDM-MAR), repB
620	*bla*_CTX-M-9_	IncA, IncFIB(K)
631	None	None
717	*bla*_OKP-B-7_	Col440II, IncFIB(K)
718	*bla*_OKP-B-7_	Col440II, IncFIB(K)
721	*bla*_SHV-89_, *floR*, *fosA*, *oqxA*, *oqxB*, *qnrS1*, *sul2*, *tet(A*)	IncR, IncX1
723	*bla*_SHV-89_, *floR*, *fosA*, *oqxA*, *oqxB*, *qnrS1*, *sul2*, *tet(A*)	IncR, IncX1
730	*bla*_CTX-M-78_, *fosA4*	IncFIB(K), IncR

### Plasmid replicon detection

PlasmidFinder detected plasmid replicon types in the isolates (Table 3). In two strains, no plasmids were observed. For other strains, various plasmid types were observed, the most frequent replicon sequences were IncFIB(K) (*n* = 12). The other types included IncR, IncX1, IncY, IncFIA(HI1), repB(R1701), pKPC-CAV1321, IncFIB(pECLA), IncFIB(pHCM2), IncFII(pECLA), IncHI1B(pNDM-MAR), and repB.

### Analysis of *sil* and *pco* gene contigs in isolates

In-depth characterization of the genetic basis of the *sil* and *pco* genes was accomplished by analyzing the WGS data against the GenBank database. A few isolate sequences yielded highly conserved matches of the *sil* gene contigs to plasmid-associated reference sequences. Three of the isolated strains (461, 462, and 496) exhibited *sil* gene contigs that were highly similar (99.85% identity) to the plasmid gene fragment from *K. pneumoniae* (CRE-243: CP128726.1) isolated in a hospital in the United States. The *sil* gene contigs in strain 476 showed a high degree of similarity to that of *Citrobacter freundii* (CFNIH9: CP026238.1; CFA1707: CP110914.1; and CF1807: CP110894.1) and *Serratia marcescens* (SJC1058: OX291724.1). Additionally, nearly 100% similarity was evident for strain 592 and *K. pneumoniae* (KP9650: CP122391.1), and for strain 607 and *K. pneumoniae* (T-hvKP: CP188907.1).

**Fig 3:**
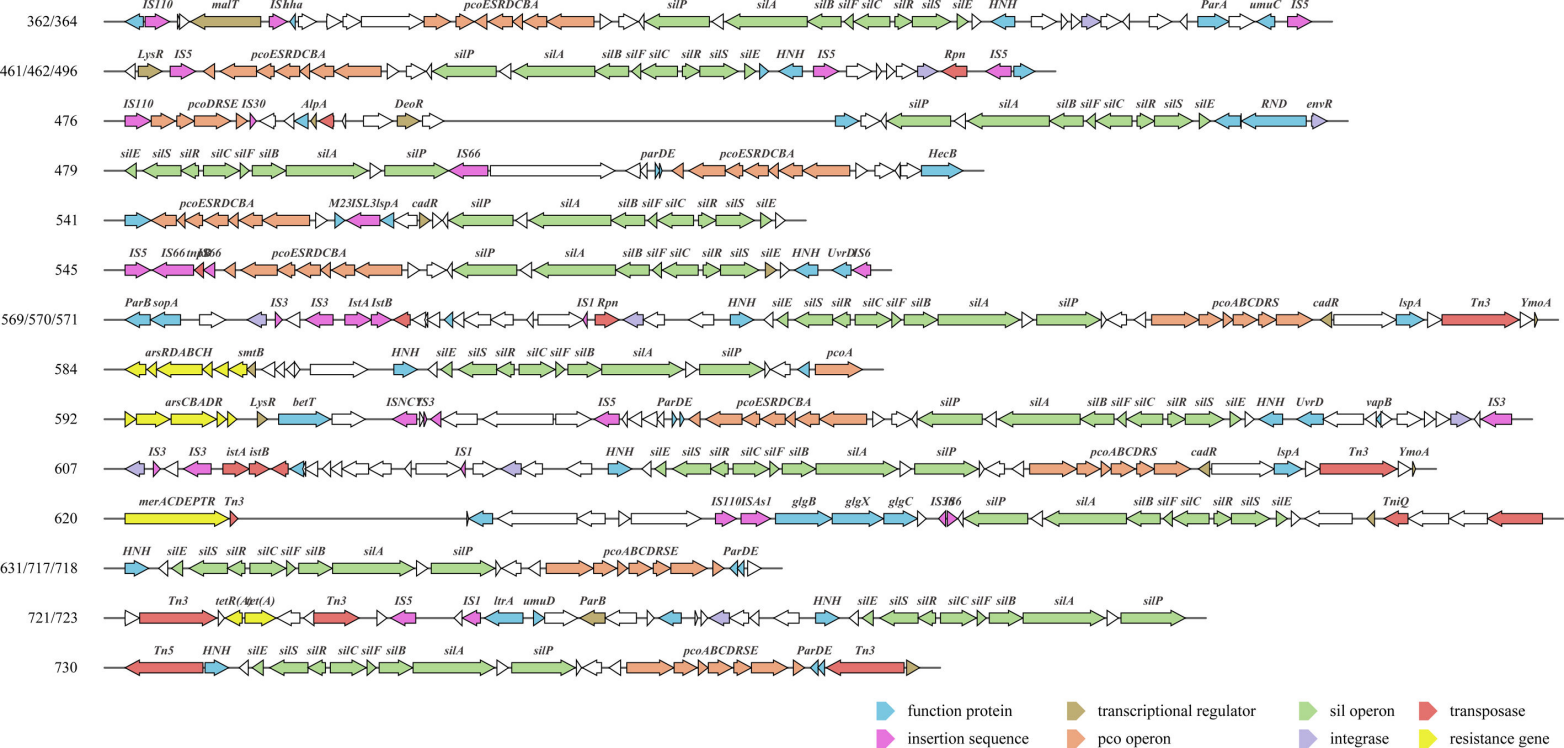
Genetic context of *sil* operon of isolates.

Based on the available sequencing data, the relevant sections of the DNA regions harboring the *sil* and *pco* genes are illustrated in Fig. 3. Based on the determined gene maps, the presence of Tn3 transposase was observed surrounding the *pco* genes in five strains. Additionally, various insertion sequence (IS) transposases were observed surrounding the *sil* genes in the isolated strains, which included IS1, IS3, IS5, IS6, IS66, and IS110. The sequencing results revealed the location of the *sil* and *pco* genes of strain 476 on different gene clusters that exhibited high similarity to gene sequences from different strains. The findings indicated that the genome of strain 476 may have undergone rearrangement.

### Clonal diversity of isolates

**Fig 4:**
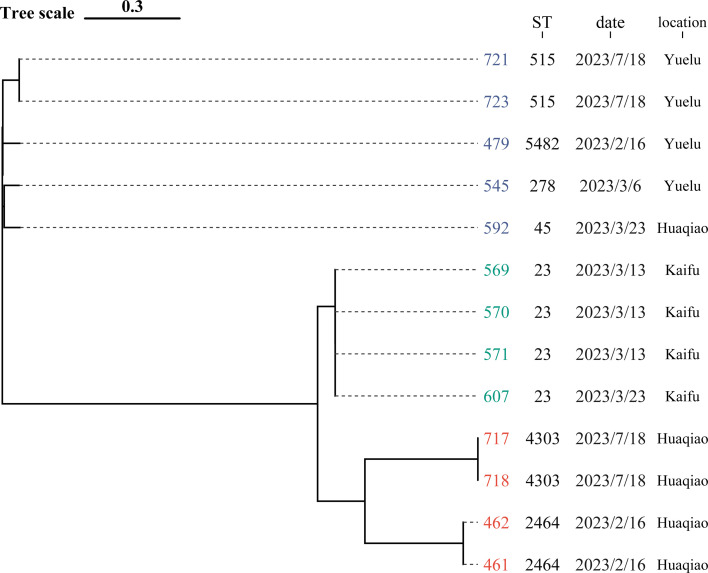
Phylogeny of *K. pneumoniae* isolates.

The multilocus sequence typing (MLST) results showed that three *E. coli* strains belonged to ST5442 (*n* = 2) and ST6701 (*n* = 1), while *K. pneumoniae* isolates belonged to ST23 (*n* = 4), ST2464 (*n* = 2), ST4303 (*n* = 2), ST515 (*n* = 2), ST45 (*n* = 1), ST278 (*n* = 1), and ST5482 (*n* = 1). Among the obtained strains, different MLST types were detected, showing a high clonal diversity of silver-resistant isolates present in the different sources. In addition, a phylogenetic tree constructed for *K. pneumoniae* strains using Roary software revealed that the 13 *K*. *pneumoniae* strains were clustered into three groups. Strains 721 and 723 were identified in one group with minor genetic variations. Strains 545 and 592, although isolated from different WWTPs and at different times, exhibited a high degree of similarity. Strains 461 and 462, and strains 717 and 718 were each separately grouped into two distinct groups. Strain 607, isolated at a different time from a different location, clustered together with strains 569, 570, and 571. Strains 718 and 717 showed high genetic similarity (Fig. 4).

## DISCUSSION

With the widespread application of silver compounds in medical and clinical settings, microbial resistance to silver has become an increasing concern. The genetic characteristics and backgrounds of various silver-resistant bacteria isolated from clinical wounds have been described. However, the issue extends beyond clinical settings; due to human activities, silver resistance has emerged in the environment, integrating into natural cycles. Detection of wastewater from WWTPs has revealed the presence of *sil* genes in multiple facilities (27). Metagenomic sequencing of both influent and effluent samples from these plants consistently identified the *sil* genes, suggesting that they may persist throughout the entire treatment process (28). This indicates that the presence of silver resistance genes in the environment is no longer a rare phenomenon. Currently, most studies focus on the concentration of Ag^+^ and silver resistance genes in wastewater environments, while only a few reports on silver-resistant bacteria in environmental settings. This is a significant research gap that needs to be addressed.

Previous studies indicated that silver-resistant strains were not widespread and were all classified within the genus *Enterobacteriaceae* (6, 29). However, comparing the MIC results of the current and previous studies is challenging due to variations in detection methods. These variations include the use of different culture media, such as MHB, salt-free Luria-Bertani broth, or IsoSensitest broth, and different MIC cut-off values (30). The choice of culture medium can significantly impact the bactericidal efficacy of Ag^+^ due to the presence of NaCl or thiol-containing components, which can precipitate or bind to Ag^+^. Furthermore, there is currently no universally accepted cut-off value for Ag^+^. Therefore, it is crucial to standardize the antimicrobial susceptibility testing procedure for Ag^+^.

All of the 22 silver-resistant strains examined harbored *sil* genes. Not surprisingly, all the strains carried the entire *sil* operon. The *sil* operon consists of multiple genes, each playing a specific role in the bacterial silver resistance mechanism. *silS* and *silR* are regulatory genes, encoding a sensor kinase and a response regulator protein, respectively, which together regulate the expression of other silver resistance genes. The *silCBA* genes encode a resistance-nodulation-cell division class cation/proton antiporter composed of three polypeptides that utilize membrane potential to pump Ag^+^ out of the cell, thereby providing resistance (31). The *silE* gene encodes a 123 amino acid periplasmic metal-binding protein that binds to Ag^+^ and reduces its toxicity (9). The *silP* gene encodes a P-type ATPase responsible for expelling Ag^+^ from the cytoplasm. The SilF protein is the periplasmic chaperone that assists in the transport and binding of Ag^+^ (32). These genes work together to form a complex silver resistance system, enabling bacteria to survive in environments containing abundant silver.

We isolated silver-resistant bacteria from the environment surrounding WWTPs. The majority of these isolates were *Klebsiella* spp., followed by *Escherichia* spp. and *Kluyvera* spp. MLST typing results revealed that among *Klebsiella* spp., there were clinically significant types, such as *Klebsiella pneumoniae* ST45, as well as highly pathogenic and virulent types, like *Klebsiella pneumoniae* ST23 (33, 34). These findings suggest a potential risk of these strains transferring to humans or animals through environmental pathways and highlight the broader risk of the spread of silver resistance genes in the environment. To investigate this further, we constructed a phylogenetic tree to analyze the genetic relationships among strains isolated at various times and locations. The results indicated that certain strains clustered together with minor variations on the phylogenetic tree, suggesting that silver resistance genes may be spreading clonally within the environment. Such clonal spread underscores the potential for these genes to persist and propagate, emphasizing the broader environmental and public health risks associated with silver resistance.

In our study, the *sil* genes of the isolated strains exhibited high sequence similarity to the reference plasmid sequences, indicating significant genetic homology between them and suggesting the possibility of horizontal gene transfer between different bacterial species and environments. Strains 461, 462, and 496 exhibit a remarkable 99.85% identity to the *sil* genes of *Klebsiella pneumoniae* (CRE-243), a hospital isolate from the United States, indicating shared genetic reservoirs between clinical and environmental strains. This shared genetic reservoir likely occurs via plasmid-mediated horizontal gene transfer. These findings suggest that clinical heavy metal resistance genes may spread to natural environments via multiple pathways. Conversely, environmental strains might act as reservoirs for resistance genes that could be reintroduced into hospital settings through water systems or direct contact. Similarly, the *sil* genes in strain 476 showed high similarity to multiple strains of *Citrobacter freundii* (CFNIH9, CFA1707, and CF1807) and *Serratia marcescens* (SJC1058), highlighting the genetic diversity and adaptability of the resistance gene across different phylogenetic species. It points to the widespread dissemination of *sil* genes within *Enterobacteriaceae*. The fact that most silver-resistant bacteria isolated in clinical settings belong to the *Enterobacteriaceae* further supports this point (6). Furthermore, the near-complete similarity of the *sil* genes in strains 592 and 607 with those of specific *K. pneumoniae* plasmids (KP9650 and T-hvKP, respectively) emphasizes the role of plasmid-mediated transfer in maintaining and propagating microbial resistance. The *sil* operon may be efficiently disseminated in environmental bacterial populations through plasmid transfer (35). Plasmids as mobile genetic elements facilitate the rapid dissemination of microbial resistance among various bacterial species through horizontal gene transfer, particularly under selective pressures such as heavy metal exposure (36).

Previous studies have identified variable incompatibility groups for *sil* gene-carrying plasmids, including IncHI2, IncFIB(K), IncHI1B, IncFII(K), and others (37–39). In the strains, the most common plasmid type was IncFIB(K). In the study by Håkonsholm et al., all *K. pneumoniae* strains carrying heavy metal tolerance genes isolated from the marine environment had their *sil* genes located on IncFIB plasmids (39). This indicates that this region is frequently observed in the IncFIB group plasmids of *K. pneumoniae*. IncFIB plasmids commonly carry multiple resistance genes and confer stability to the genes integrated into the plasmid (40, 41). In our study, WGS confirmed that the plasmid types in isolates with the *sil* operon were variable, including IncFIB, IncR, IncFIA, repB, IncA, and IncHI1B, which is consistent with previous findings. From the identified gene maps, it is apparent that multiple transposases may be involved in the transfer of the *sil* cassette to different plasmids and the chromosome. Further monitoring and genome data are necessary to assess the impact of either plasmid-associated or chromosome-associated silver resistance on human, animal, and environmental health.

Unlike silver-resistant bacteria isolated from clinical settings, the strains isolated in our study were predominantly sensitive to antibiotics. Strains 362 and 364 harbored *sul3* and *dfrA12*, together with strains 592, 721, and 723, which harbored *sul2* and *floR*, with trimethoprim/sulfamethoxazole resistance phenotype. Strains 362 and 364 harbored *tet(B*), and strains 461, 462, 592, 721, and 723 harbored *tet(A*). These strains did not exhibit tigecycline resistance, indicating that the genes have not undergone mutations that confer tigecycline resistance. The long-term presence of heavy metals in the environment exerts strong and persistent selective pressure on microorganisms, leading to increased presence of antibiotic resistance genes (42). In our study, different combinations of extended-spectrum *β*-lactamase and *sil* genes were observed. Co-occurrence of CTX-M and silver resistance genes was previously detected in one study performed among isolates from human and bird samples in Sweden and another from the polluted stretch of river Yamuna from India (43, 44). It is worth noting that strain 620 displayed resistance to colistin, even though no *mcr* genes were detected. Consequently, through genetic comparison, we identified mutations in the *phoP* and *phoQ* genes in strain 620, which have led to changes in its resistance to colistin. A previous study demonstrated the potential of Ag^+^ as a broad-spectrum inhibitor for the treatment of *mcr*-positive bacterial infections in combination with colistin (45). Hence, silver is typically used as the last line of defense in treating colistin-resistant superbugs. Şirin et al. (46) demonstrated that nanostructured antimicrobials obtained by conjugating colistin and meropenem antibiotics with biosynthesized silver nanoparticles had efficient antibacterial and antibiofilm properties against MDR *E. coli* and *K. pneumoniae* strains. However, the identification of bacteria with dual resistance to silver and colistin indicates that the use of silver-based agents to treat colistin-resistant bacteria is limited. Although this phenomenon remains rare, it is a dangerous tendency and should be closely monitored.

In our study, we examined bacterial strains displaying resistance to silver-rich environments. These strains, which harbored multiple *sil* genes, harbored the *cus* operon. In bacteria like *E. coli*, the cus gene system senses extracellular Ag^+^ and modulates gene expression to enhance resistance. The *cusR* and *cusS* genes within the *cus* gene system play a crucial role in regulating this process. They help resistant strains adapt to the stress of Ag^+^ by controlling the expression of downstream genes. In a silver-resistant strain of *E. coli* obtained through exposure to increased silver concentrations, induction of chromosomal genes encoding protein CusB and CusF (and possibly CusA or CusC) was observed, accompanied by a mutation in the *cusS* gene (47). In addition to the *cus* genes, sequencing results indicated that the isolated strain carried the *pco* genes. The proteins encoded by the *pco* genes were capable of transporting Ag^+^ from the inside to the outside of cells, thereby reducing the intracellular concentration of Ag^+^. This expulsion mechanism requires energy and typically relies on ATP-binding cassette transport proteins located in the cell membrane. The expression of the *pco* genes might be induced by the concentration of Ag^+^, thereby upregulating their expression in the presence of Ag^+^ and enhancing the silver resistance of the bacteria. However, a few isolates lacked the *pcoE* gene. A previous study indicated that with a high affinity to both copper ions and Ag^+^, *pcoE*, encoded in the *pco* operon, could function as a metal sponge for these ions in the bacterial periplasm before the induction of main *pco* system proteins (48). Similar properties and structures have been reported for the homologous silver-binding protein SilE (31). These genes all contributed to the survival of bacteria in silver-containing environments.

WGS revealed that all strains isolated in our study concurrently harbor multiple heavy metal resistance genes. Among these, the *ars* genes were most predominant. The *mer* genes, associated with resistance to mercury, and the *znt* genes, linked to resistance to zinc, were prevalent. These observations suggest that a diverse array of heavy metal resistance genes is already present in the ambient environment surrounding WWTPs. Yuan et al. (49) investigated the abundance of mercury and silver resistance genes in WWTPs. Their findings indicate that these facilities act as significant reservoirs of heavy metal resistance genes. The development of bacterial resistance to heavy metals is a multifaceted phenomenon driven by a complex array of genetic determinants operating at the molecular level. Key mechanisms involved in this process include transportation, biosorption, and co-metabolism/redox reactions, which together contribute to the intricate network of resistance mechanisms deployed by bacteria in response to heavy metal exposure (50). Cai et al. (51) isolated strains carrying multiple heavy metal tolerance genes from wastewater, while Zagui et al. (52) similarly identified strains harboring heavy metal tolerance genes during the analysis of hospital and urban wastewater treatment plant effluents, with the *silA* and *pcoD* genes being the most common. Comparing our findings with those from other studies confirms that, at present, heavy metals generated by human activities cannot be completely removed in WWTPs and exert selection pressure, thereby promoting the development of heavy metal resistance in bacteria. Therefore, the use of silver should be reduced and restricted, and efforts should continue to improve the ability to remove silver and silver-resistant genes.

In summary, silver-resistant bacteria are widely present in the environment surrounding WWTPs. These strains carrying the *sil* operon typically harbor multiple heavy metal resistance genes, including *mer* and *ars*. These findings indicate that urban WWTPs cannot completely eliminate the influence of heavy metals presently, with the ensuing contamination of surrounding water bodies. Efforts should be made to limit the use of silver, enhance WWTP purification methods, reduce environmental and human health risks, and prevent the further spread of silver resistance.

### Conclusion

Our study emphasizes the epidemiological characteristics of silver-resistant bacteria in the environments surrounding WWTPs. Silver resistance is conferred through both endogenous and exogenous mechanisms, with the *sil* operon playing a key role in exogenous resistance. We observed that silver resistance genes frequently coexist with multiple heavy metal resistance genes in these isolates, with the majority being plasmid-mediated, suggesting clonal spread within the environment. Moreover, the majority of isolated silver-resistant bacteria were common clinical pathogens, some of which were classified as highly pathogenic and virulent types. Infections caused by these strains render silver-based treatments less effective. Currently, silver and silver resistance genes in wastewater cannot be completely eliminated, thereby promoting the development of silver resistance in bacteria. Therefore, focusing on restricting the use of silver, improving WWTP purification methods, and implementing measures to prevent the further dissemination of silver resistance is essential. These actions are crucial to protect both environmental and human health.

## Data Availability

The genome data sequenced in this study are available in GenBank under accession no. PRJNA1271949. Data will be made available on request.

## SUPPLEMENTAL MATERIAL

Supplemental material aem.00022-25-s0001.pdf Tables S1 to S3; Fig. S1.

ASM does not own the copyrights to Supplemental Material that may be linked to, or accessed through, an article. The authors have granted ASM a non-exclusive, world-wide license to publish the Supplemental Material files. Please contact the corresponding author directly for reuse.
