# Supplementary material for: Whole-genome sequencing characterization of silver-resistant bacteria from the outfall of wastewater treatment plants and effluent-receiving rivers
Source: Appl Environ Microbiol. 2025 Aug 5;91(9):e00022-25. doi: 10.1128/aem.00022-25 (PMC12442373; doi:10.1128/aem.00022-25)

Supplemental material

Table S1 Primers Used For *sil* Genes And *pco* Genes Detection with The Expected Length

Gene	Forward Primer (5'-3')	Reverse Primer (5'-3')	Length(bp)
silE	AGGGGAAACGGTCTGACTTC	ATATCCATGAGCGGGTCAAC	221
silF	CGATATGAATGCTGCCAGTG	ATTGCCCTGCTGAATAAACG	229
silP	CCTGGGTTTACAGCGTCATT	ATGGCACCTGAGGTTTGTTC	172
silCBA	CGGGAAACGCTGAAAAATTA	GTACGTTCCCAGCACCAGTT	189
silRS	GGCAATCGCAATCAGATTTT	GTGGAGGATACTGCGAGAGC	189
pcoA	CGGCCAGGTTCACGTCCGTC	TGCCAGTTGCCGCATCCCTG	1673
pcoB	TCGGCAATTTACAGGGGCAT	GCAGGTCTGAAATCCTGCCT	274
pcoC	TTCTTACAGGTGGCCTCGTT	CCGGTAATAGGGTGCGTATC	334
pcoD	CAGGAACGGTGATTGTTGTA	CCGTAAAATCAAAGGGCTTA	702
pcoR	CAGGTCGTTACCTGCAGCAG	CTCTGATCTCCAGGACATATC	636
pcoS	GGGATGTTTCGCATTACCGC	CTGGGCTGCAGAGACAATGA	342
pcoE	CTGGGCTGCAGAGACAATGA	TCCGCCTGTTGCTGATGAAT	351

Table S2 The Isolate Numbers and Their Corresponding Species

Number	Species	Source	Number	Species	Souce
362	*Escherichia coli*	water	571	*Klebsiella pneumoniae*	water
364	*Escherichia coli*	water	584	*Escherichia coli*	solid
461	*Klebsiella pneumoniae*	water	592	*Klebsiella pneumoniae*	water
462	*Klebsiella pneumoniae*	water	607	*Klebsiella pneumoniae*	water
476	*Citrobacter freundii*	water	620	*Kluyvera ascorbata*	solid
479	*Klebsiella pneumoniae*	water	631	*Kluyvera ascorbata*	solid
496	*Klebsiella variicola*	water	717	*Klebsiella pneumoniae*	water
541	*Enterobacter cloacae*	water	718	*Klebsiella pneumoniae*	water
545	*Klebsiella pneumoniae*	solid	721	*Klebsiella pneumoniae*	water
569	*Klebsiella pneumoniae*	water	723	*Klebsiella pneumoniae*	water
570	*Klebsiella pneumoniae*	water	730	*Enterobacter asburiae*	water

Table S3 Antimicrobial susceptibility profiles of silver resistant isolates

Isolates	TGC	COL	CIP	MEM	CFX	FEP	IPM	CAZ	CRO	LEV	AK	SXT	TZP	CN	ATM
362	0.25	1	≤0.125	≤0.125	4	≤0.125	≤0.125	≤0.125	≤0.125	0.5	8	4/76	2/4	1	≤0.125
364	0.25	0.5	≤0.125	≤0.125	16	≤0.125	≤0.125	0.25	≤0.125	0.5	4	4/76	2/4	1	≤0.125
461	0.5	0.5	≤0.125	≤0.125	4	≤0.125	0.25	0.25	≤0.125	0.25	4	0.5/9.5	1/4	1	≤0.125
462	0.5	0.5	≤0.125	≤0.125	2	≤0.125	≤0.125	0.25	≤0.125	≤0.125	4	0.5/9.5	2/4	1	≤0.125
476	0.5	0.5	≤0.125	≤0.125	/*	≤0.125	0.25	0.5	0.25	≤0.125	4	≤0.125/2.375	2/4	0.5	≤0.125
479	0.5	0.5	≤0.125	≤0.125	2	≤0.125	≤0.125	≤0.125	≤0.125	≤0.125	4	0.25/4.75	1/4	1	≤0.125
496	0.5	0.5	≤0.125	≤0.125	4	≤0.125	≤0.125	≤0.125	≤0.125	≤0.125	4	0.25/4.75	2/4	1	≤0.125
541	1	0.5	≤0.125	≤0.125	/	≤0.125	0.5	0.5	0.5	≤0.125	4	≤0.125/2.375	2/4	1	≤0.125
545	0.5	0.5	≤0.125	≤0.125	2	≤0.125	≤0.125	≤0.125	≤0.125	≤0.125	4	0.25/4.75	4/4	1	≤0.125
569	0.5	0.5	≤0.125	≤0.125	16	0.25	≤0.125	0.5	0.25	≤0.125	4	0.5/9.5	4/4	1	≤0.125
570	0.5	1	≤0.125	≤0.125	4	≤0.125	0.25	0.25	≤0.125	≤0.125	4	0.5/9.5	4/4	1	≤0.125
571	0.5	1	≤0.125	≤0.125	4	≤0.125	0.25	0.25	≤0.125	≤0.125	4	0.5/9.5	1/4	1	≤0.125
584	0.25	0.25	≤0.125	≤0.125	8	≤0.125	≤0.125	≤0.125	≤0.125	≤0.125	8	≤0.125/2.375	1/4	1	≤0.125
592	1	1	0.5	≤0.125	4	≤0.125	≤0.125	≤0.125	≤0.125	0.5	4	4/76	4/4	1	≤0.125
607	0.5	1	≤0.125	≤0.125	16	≤0.125	0.25	0.25	≤0.125	≤0.125	4	1/19	4/4	1	≤0.125
620	0.5	16	≤0.125	≤0.125	16	≤0.125	0.25	0.25	2	0.25	8	≤0.125/2.375	4/4	1	≤0.125
631	1	1	≤0.125	≤0.125	8	0.25	0.5	0.5	0.5	≤0.125	8	≤0.125/2.375	4/4	4	≤0.125
717	0.5	0.5	≤0.125	≤0.125	4	≤0.125	0.25	≤0.125	≤0.125	≤0.125	4	0.5/9.5	1/4	1	≤0.125
718	1	1	≤0.125	≤0.125	4	≤0.125	0.25	≤0.125	≤0.125	≤0.125	4	0.5/9.5	1/4	1	≤0.125
721	1	1	1	≤0.125	4	≤0.125	0.25	≤0.125	≤0.125	1	4	4/76	2/4	1	≤0.125
723	1	1	1	≤0.125	4	≤0.125	0.25	≤0.125	≤0.125	1	8	4/76	2/4	1	≤0.125
730	0.5	0.5	≤0.125	≤0.125	/	≤0.125	2	≤0.125	≤0.125	0.5	4	0.5/9.5	1/4	1	≤0.125

Note: *Naturally resistant to this antibiotic.

Fig. S1 The PCR results of strain 461

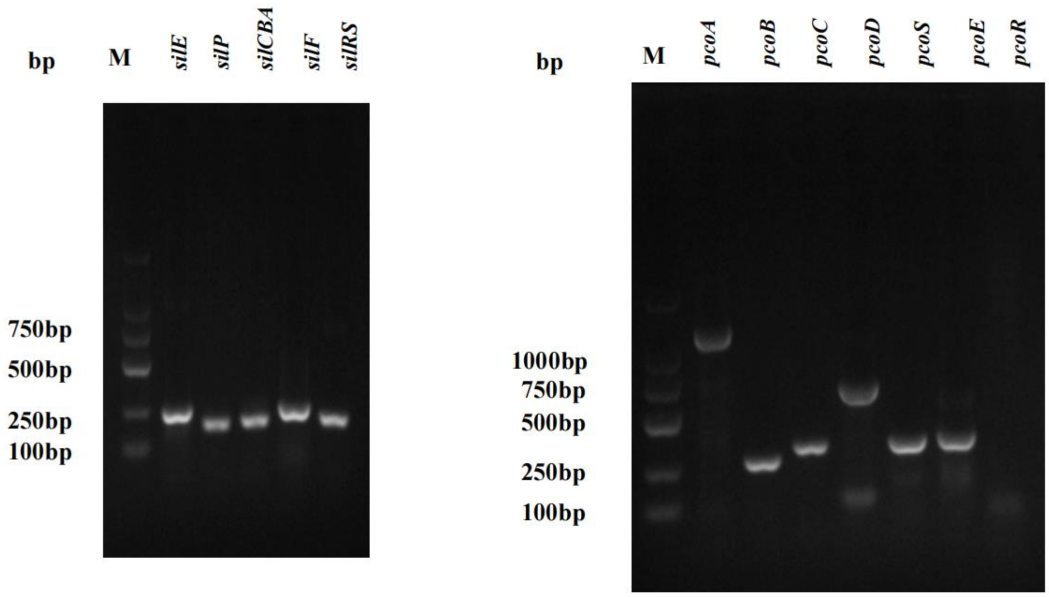

Supplement: Supplemental material — Tables S1 to S3; Fig. S1. [file aem.00022-25-s0001.pdf]
